# Association of estimated glucose disposal rate with atrial fibrillation, heart failure and cardiovascular mortality in patients with diabetes: a prospective cohort study from the UK Biobank

**DOI:** 10.3389/fendo.2025.1579836

**Published:** 2025-07-18

**Authors:** Zhen Tan, Yijun Liu, Lei Liu, Shuang Li, Xinrui Xue, Xiaoping Li, Hongqiang Ren

**Affiliations:** ^1^ Department of Cardiology, Suining Central Hospital, Suining, Sichuan, China; ^2^ Department of Cardiology, Sichuan Provincial People’s Hospital, University of Electronic Science and Technology of China, Chengdu, Sichuan, China

**Keywords:** estimated glucose disposal rate, cardiovascular disease, atrial fibrillation, heart failure, cardiovascular mortality, patients with diabetes, UK Biobank

## Abstract

**Background:**

Estimated glucose disposal rate (eGDR) was a novel non-insulin-based marker of insulin resistance (IR), which had been used in many studies to evaluate the clinical prognosis of diabetes. However, the association of eGDR with atrial fibrillation (AF), heart failure (HF) and cardiovascular mortality in patients with diabetes remains unclear.

**Methods:**

The study utilized UK Biobank data from 31,733 participants. Kaplan-Meier curves and Log-rank tests assessed AF, HF, and cardiovascular mortality incidence. Multivariate Cox models and restricted cubic splines analyzed the associations of eGDR with these outcomes. Polygenic Risk Score (PRS) analysis evaluated the joint effects of eGDR and PRS. Boruta algorithm filtered key predictive variables. Subgroup analysis was performed using cardiovascular high-risk factors, and mediation analysis explored the relationships of eGDR with the outcomes.

**Results:**

Subjects with higher eGDR were more likely to be female, younger, more physically active, non-smoker, and non-drinker. The cumulative incidence of AF, HF, and cardiovascular mortality in the higher quartiles of GDR were significantly lower than those in the lowest quartile (log-rank *P* < 0.001 for all). eGDR exhibited an independent negative linear correlation with the risk of AF (HR = 0.94, 95% CI: 0.91-0.96), HF (HR = 0.78, 95% CI: 0.74-0.82), and cardiovascular mortality (HR = 0.86, 95% CI: 0.83-0.88) risk. eGDR made the most significant contribution to the predicted outcomes. In diabetic patients with high genetic susceptibility, high eGDR could reduce the risk of AF (HR = 0.68, 95% CI: 0.51-0.90), HF (HR = 0.43, 95% CI: 0.29-0.62), and cardiovascular mortality (HR = 0.30, 95% CI: 0.22-0.42). Mediation analysis demonstrated that 10.7%, 7.9%, and 10.3% of the relationship between eGDR and AF, HF, and cardiovascular mortality among individuals with diabetes were mediated by eGFR, respectively.

**Conclusions:**

This study demonstrated that higher eGDR levels were associated with a decreased risk of AF, HF, and cardiovascular mortality. Therefore, eGDR may serve as a valuable tool for predicting the risk of AF, HF, and cardiovascular mortality in patients with diabetes.

## Introduction

Diabetes, a chronic metabolic disorder characterized by persistently elevated blood glucose levels, has emerged as a significant global health burden, ranking among the leading causes of death and disability worldwide (1). Individuals with diabetes exhibit a markedly increased risk of cardiovascular disease (CVD), which encompasses myocardial infarction (MI), heart failure (HF), atrial fibrillation (AF), and cardiovascular mortality (2, 3). Identifying risk factors for CVD in diabetic patients is critical, prompting extensive research into various biomarkers that can assess CVD risk in both diabetic and non-diabetic populations (4–7).

The estimated glucose disposal rate (eGDR) is an index derived from waist circumference, hypertension, and glycated hemoglobin (HbA1c), used to evaluate the body’s capacity to process glucose (8). Notably, eGDR is closely linked to insulin resistance (IR), a key risk factor for CVD (9). IR not only underpins type 2 diabetes but also contributes to a cluster of metabolic abnormalities, including dyslipidemia, inflammation, and endothelial dysfunction, all of which drive the onset and progression of CVD (10). Consequently, eGDR has increasingly been utilized as a predictor of cardiovascular mortality risk (11–13). Traditional methods for assessing insulin sensitivity, such as the glucose clamp technique, are often complex, invasive, time-consuming, expensive, and require specialized equipment and trained personnel (14). In contrast, eGDR offers a simple, convenient, and cost-effective alternative, as it can be readily calculated using routinely available clinical parameters. This makes eGDR particularly suitable for routine clinical practice and large-scale epidemiological studies (15, 16).

Previous studies have demonstrated that eGDR is independently and negatively correlated with coronary artery disease severity, suggesting its potential utility in early identification and risk stratification of coronary heart disease patients, thereby improving prognosis (17). Similarly, eGDR has been shown to predict cardiovascular events and mortality in non-diabetic patients with chronic kidney disease (18). Additionally, eGDR has been associated with arterial stiffness and mortality in adults with non-alcoholic fatty liver disease (19). However, evidence regarding the association between eGDR and AF, HF, and cardiovascular mortality remains limited. Given that AF and HF represent two major cardiovascular complications of diabetes, elucidating their relationship with eGDR could provide novel insights into the pathophysiology of these conditions and inform more effective preventive and therapeutic strategies. Therefore, the aim of this study was to evaluate the association of eGDR with AF, HF, and cardiovascular mortality in patients with diabetes and to explore the underlying mechanisms.

## Materials and methods

### Study population

The UK Biobank (UKB) is a large-scale biomedical database and research resource and has collected an unprecedented amount of biological and medical data on 500,000 participants (229,041 males and 273,293 females) from UK. UKB has received approval from the North West Multi-centre Research Ethics Committee (MREC) as a Research Tissue Bank (RTB) approval. Therefore, researchers do not require separate ethical clearance and can operate under the RTB approval. Data from the UKB are accessible to researchers after receiving research approval from the UKB. This study was conducted under UKB licence (Application ID:106027).

This study sifted 36,126 participants who had evidence of diabetes at baseline, the inclusion criteria were as follows: (1) Hospital diagnosis records indicating diabetes. (2) HbA1c ≥ 6.5%. (3) Fasting glucose ≥ 7.0mmol/L. (4) Receipt of hypoglycemic treatment. Participants were excluded if they had missing data on waist circumference (WC), glycosylated hemoglobin (HbA1c), or a history of AF and HF. As a result, a total of 31,733 participants were included in the final analysis.

### Data collection and definition

At recruitment, participants completed computerised questionnaires on lifestyle, baseline demographic, and medical history, including gender, age, race, education level, body mass index (BMI), WC, height, smoking status (never, former, and current), alcohol consumption status (never, former, and current), frequency of moderate physical activity (Never, < 3 times per day, ≥ 3 times per day), household income. Hypertension was defined as systolic blood pressure (SBP) ≥ 140 mmHg, diastolic blood pressure (DBP) ≥ 90 mmHg, a hospital diagnosis record, use of blood pressure medication, specialist diagnosis, drug reimbursement records, or self-reported information. Additional data collected included the use of aspirin, insulin, blood pressure medication, and cholesterol-lowering medication. Laboratory assessments included measurements of glycated hemoglobin (HbA1c), glucose (Glu), albumin (ALB), serum creatinine (Scr), uric acid (Ua), blood urea nitrogen (BUN), triglycerides (TG), total cholesterol (TC), high-density lipoprotein cholesterol (HDL-C), low-density lipoprotein cholesterol (LDL-C), estimated glomerular filtration rate (eGFR), and C-reactive protein (CRP).

The formula used to calculate the eGDR was as follows: eGDR=21.158 - [(0.09 × waist circumference (cm)] - [(3.407 × Hypertension (yes or no)] - [0.551 × HbA1c (%)] (15). Participants were categorized into four groups (Q1, Q2, Q3, Q4) based on eGDR level using the 25th, 50th, and 75th percentiles as cutoff points. The lowest quartile (Q1) served as the reference group.

### Assessment of atrial fibrillation, heart failure and cardiovascular mortality

Hospital records linked to disease outcomes were obtained from the UK Biobank. The international statistical classification of diseases (ICD-10) was used to define the classification of diseases. The primary endpoints of this study were the diagnosis of AF (codes I48, I48.0-4, and I48.9), HF (codes I50, I13.0, I13.2, and I11.0) and cardiovascular death. Cardiovascular death was defined based on records as mortality caused by acute myocardial infarction (AMI), AF, HF, arrhythmia, cardiac surgery, or other cardiovascular-related causes. For this study, the hospital admission and death data were updated up to 19 December 2022, and thus the follow-up period was terminated on this date. The date of diagnosis was defined as the earliest day when the disease manifested. The follow-up duration was calculated as the time interval between the baseline survey date and the earliest occurrence among the disease diagnosis date, the death date, the loss-to-follow-up date, or the end of the follow-up period. During the follow-up period, cardiovascular mortality rates were computed for each quartile of eGDR.

### Definition of polygenic risk score

Patients with missing Polygenic Risk Score (PRS) data were excluded from the analysis. Consequently, a total of 31,375 diabetic patients were included in the PRS analysis. Standard PRS for AF and CVD, available from the UK Biobank, has been previously published. The PRS was calculated as the weighted sum of the effect sizes of individual genetic variants multiplied by their allele dosages, using a Bayesian approach applied to meta-analyze summary statistics derived from genome-wide association study (GWAS) data (20). In this study, the PRS of AF and CVD were categorized into low genetic risk (quintile 1), intermediate genetic risk (quintile 2 to 4) and high genetic risk (quintile 5).

### Statistical analysis

R software (version 4.3.0, Institute for Statistics and Mathematics, Vienna, Austria) was used for all analyses. Continuous variables were expressed as mean ± standard deviation, and if variables were normally distributed, analysis of variance (ANOVA) was performed to assess differences between groups. Otherwise, they were presented as Median or quartile *M* (P25, P75). Mann Whitney U test was used for comparison among quartiles. Categorical variables were expressed as frequency and percentage. Chi-square tests were conducted to compare proportions across groups. Missing values of covariates were handled using multiple imputation. The maximum proportion of missing values was 6.1%, with an average of 0.51%.

The cumulative incidence rate curves for AF, HF, and cardiovascular mortality were estimated using the Kaplan-Meier method and compared using the log-rank test across eGDR quartile groups. Three multivariate Cox proportional hazards regression models were constructed to evaluate the the association of eGDR with AF, HF and cardiovascular mortality in patients with diabetes. Model 1 was unadjusted; model 2 adjusted for age, gender, race, education level, BMI, smoking status, and alcohol consumption status. Model 3 included all variables from Model 2, and further adjusted for SBP, DBP, TG, TC, eGFR, UA, aspirin, cholesterol-lowering medication, blood pressure medication, and insulin use. Nonlinear correlations between eGDR and AF, HF and cardiovascular mortality were explored using a restricted cubic spline (RCS) curve based on Cox regression model.

To evaluate the joint effects of PRS on the association of eGDR with risk of AF, HF and cardiovascular mortality, analyses were stratified by genetic risk categories (low genetic risk, intermediate genetic risk and high genetic risk). Each genetic risk category was further divided into four groups (Q1, Q2, Q3, Q4) based on eGDR level using the 25th, 50th, and 75th percentiles as cutoff points individually. The association of eGDR with the risk of AF, HF, and cardiovascular mortality was analyzed using multivariate Cox proportional hazards regression Model 3.

Subgroup analyses were conducted for significant covariates (age, gender, BMI, education level, smoking status, and alcohol consumption status) to assess the effects of eGDR on the incidence of AF, HF, and cardiovascular mortality across several subgroups. We used the Random Forest-based Boruta`s algorithm for feature selection to filter out the variables that contribute most to prediction model by generating shadow features and comparing their importance. Mediation analysis was performed to investigate the mediating effects of eGDR on AF, HF, and cardiovascular mortality. Variables in Model 3 that exhibited substantial relationships with eGDR were selected as potential mediators. The percentage mediated was computed as indirect effect/(indirect+direct impact). A two-sided *P* values<0.05 indicated statistical significance.

## Result

### Demographic characteristics of the study participants

**Figure 1:**
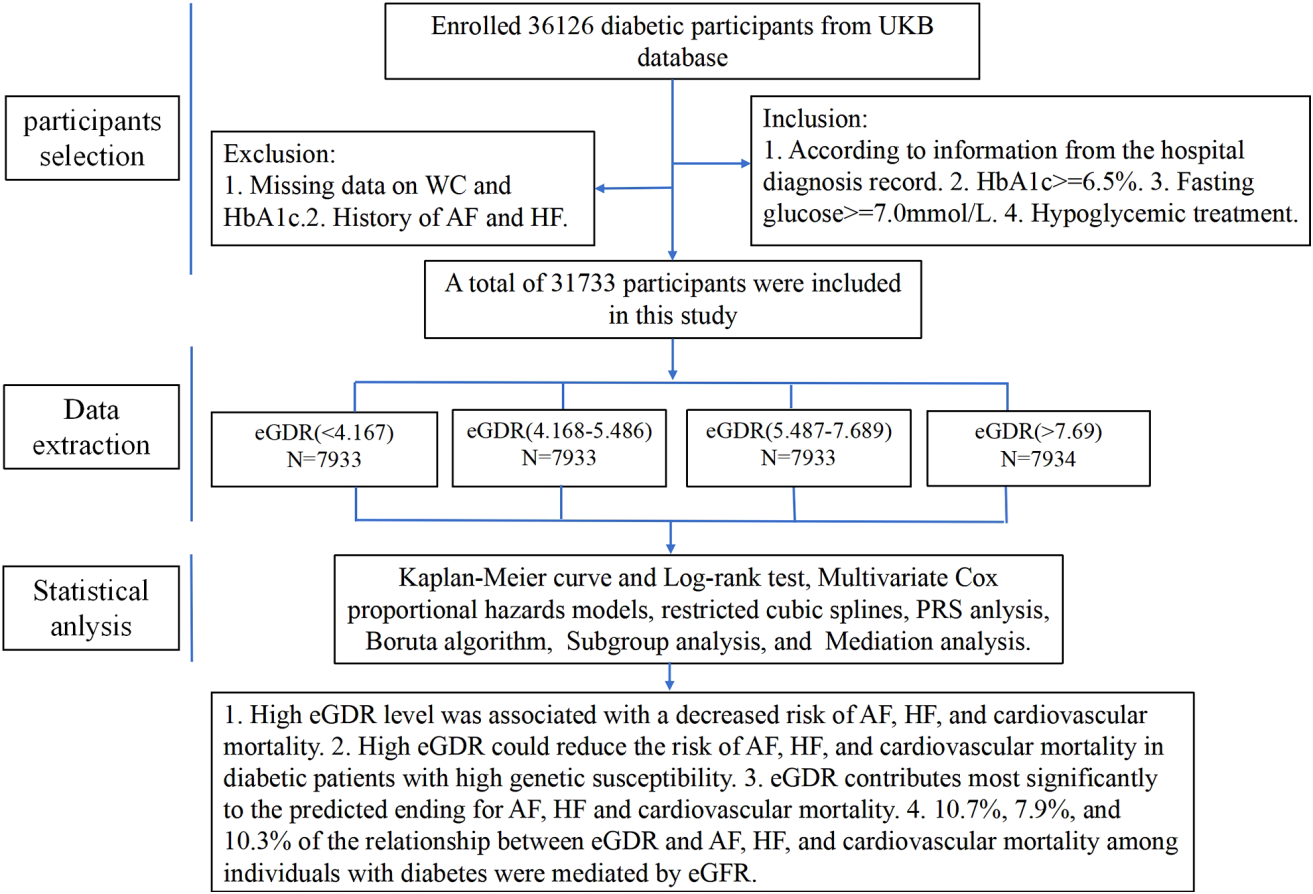
Flow chart of study selection.

A total of 31,733 diabetes patients without AF and HF were included in this study from the UKB finally, and the patient selection process was shown in **Figure 1**. Demographic characteristics of the subjects according to the quartiles of eGDR were showed in **Table 1**. The average age of the subjects was 59.192 ± 7.295 years, with a majority being non-Hispanic White (88.56%). The mean value of eGDR was 5.934 ± 2.532. Subjects with higher eGDR were more likely to be female, younger, more physically active, non-smoker, and non-drinker. Additionally, subjects with atrial fibrillation, heart failure, or cardiovascular death exhibited lower eGDR levels. The group with the highest eGDR exhibited the lowest proportion of patients using blood pressure medication, cholesterol-lowering medication, aspirin, and insulin.

**Table 1:** Demographic characteristics of the study participants.

Characteristic	Total	eGDR				P
		Q1 (<4.167)	Q2 (4.168-5.486)	Q3 (5.487-7.689)	Q4 (>7.690)	
Participants	n=31733	n=7933	n=7933	n=7933	n=7934	
Age, years	59.192±7.295	59.035±6.949	60.328±6.681	59.825±7.151	57.58±8.032	<0.001
Gender, male, n (%)	18178 (57.28)	5548 (69.94)	5135 (64.73)	4182 (52.72)	3313 (41.76)	<0.001
BMI, kg/m2	30.869±5.855	36.374±5.676	31.239±4.112	28.889±4.653	27.003±4.145	<0.001
Height, cm	2.5±1.54	2.234±1.468	2.422±1.516	2.559±1.551	2.785±1.573	<0.001
WC, cm	100.97±14.733	116.861±11.023	102.868±7.121	94.987±11.647	89.183±11.381	<0.001
SBP, mmHg	145.788±18.696	149.217±18.336	149.184±18.431	147.676±18.728	137.005±16.359	<0.001
DBP, mmHg	84.006±10.395	86.904±10.886	85.651±10.616	84.189±10.21	79.242±7.924	<0.001
HbA1c, (%)	6.812±1.357	7.704±1.66	6.767±0.995	6.51±1.193	6.268±1.007	<0.001
Hypertention, n (%)	22195 (69.94)	22195 (69.94)	22195 (69.94)	22195 (69.94)	22195 (69.94)	<0.001
eGDR	5.934±2.532	3.007±1.029	4.839±0.375	6.387±0.624	9.501±1.167	<0.001
Education level, n (%)						<0.001
College or above	7798 (25.05)	1681 (21.62)	1720 (22.08)	1922 (24.77)	2475 (31.72)	
others	23330 (74.95)	6093 (78.38)	6071 (77.92)	5838 (75.23)	5328 (68.28)	
Race						<0.001
White	27892 (88.56)	7204 (91.42)	6895 (87.44)	6862 (87.30)	6931 (88.06)	
Latin	202 (0.64)	48 (0.61)	48 (0.61)	60 (0.76)	46 (0.58)	
Black	2512 (7.98)	425 (5.39)	667 (8.46)	716 (9.11)	704 (8.94)	
Asian	890 (2.83)	203 (2.58)	275 (3.49)	222 (2.82)	190 (2.41)	
Smoking status, n (%))						<0.001
Never	14978 (47.67)	3309 (42.09)	3536 (45.04)	3916 (49.89)	4217 (53.69)	
Former	12918 (41.12)	3676 (46.76)	3518 (44.81)	3048 (38.83)	2676 (34.07)	
Now	3521 (11.21)	877 (11.15)	797 (10.15)	886 (11.29)	961 (12.24)	
Alcohol consumption status, n (%)						<0.001
Never	2631 (8.33)	619 (7.84)	646 (8.18)	671 (8.51)	695 (8.82)	
Former	2084 (6.60)	675 (8.55)	514 (6.51)	457 (5.79)	438 (5.56)	
Now	26854 (85.06)	6604 (83.62)	6741 (85.32)	6760 (85.70)	6749 (85.63)	
Frequency of moderated physical activity, n (%)						<0.001
Never	5278 (18.29)	1771 (24.85)	1294 (18.02)	1190 (16.43)	1023 (14.00)	
<3 times per day	10239 (35.48)	2494 (34.99)	2585 (35.99)	2525 (34.87)	2635 (36.06)	
≥3 times per day	13342 (46.23)	2862 (40.16)	3303 (45.99)	3527 (48.70)	3650 (49.95)	
Medications						
Cholesterol-lowering medication, n (%)	18997 (60.17)	5673 (71.78)	5614 (71.04)	4720 (59.85)	2990 (37.94)	<0.001
Blood pressure medication, n (%)	17029 (53.93)	6280 (79.46)	6050 (76.55)	4454 (56.47)	245 (3.11)	<0.001
Aspirin, n (%)	12773 (40.47)	4036 (51.13)	3864 (48.92)	3094 (39.22)	1779 (22.59)	<0.001
Insulin, n (%)	18997 (60.17)	5673 (71.78)	5614 (71.04)	4720 (59.85)	2990 (37.94)	<0.001
Laboratory assessments						
Scr, umol/L	74.125±27.087	77.247±32.252	76.797±30.257	73.315±26.105	69.236±16.291	<0.001
eGFR, ml/min	87.96 (76.785-98.247)	85.306 (72.443-96.657)	85.961 (74.577-96.143)	87.907 (77.351-97.683)	92.149 (82.153-101.297)	<0.001
Ua, umol/L	329.089±84.511	349.869±89.019	345.259±81.717	324.739±80.827	297.131±75.625	<0.001
BUN, umol/L	5.732±1.813	5.954±2.161	5.871±1.91	5.695±1.68	5.415±1.369	<0.001
TC, mmol/L	4.858±1.215	4.608±1.143	4.68±1.146	4.92±1.235	5.215±1.239	<0.001
TG, mmol/L	1.870 (1.294-2.69)	2.175 (1.561-3.05)	1.968 (1.407-2.77)	1.772 (1.233-2.545)	1.568 (1.071-2.312)	<0.001
LDL-C, mmol/L	1.414 (1.153-1.728)	1.34 (1.097-1.625)	1.375 (1.132-1.656)	1.423 (1.161-1.756)	1.532 (1.242-1.856)	<0.001
HDL-C, mmol/L	1.184 (1.001-1.422)	1.069 (0.919-1.241)	1.144 (0.981-1.339)	1.234 (1.05-1.472)	1.338 (1.105-1.62)	<0.001
Cys-C, mmol/L	0.927 (0.832-1.048)	0.984 (0.872-1.125)	0.951 (0.854-1.071)	0.919 (0.827-1.031)	0.873 (0.791-0.97)	<0.001
GLU, mmol/L	7.044 (5.466-8.56)	7.678 (5.933-10.648)	6.616 (5.366-8.206)	6.678 (5.307-8.016)	7.104 (5.411-8.045)	<0.001
ALB, g/L	44.9±2.85	44.331±2.884	45.181±2.82	45.169±2.835	44.918±2.779	<0.001
CRP, g/L	1.93 (0.92-4.02)	2.98 (1.5-6.06)	1.98 (0.97-3.9)	1.66 (0.8-3.49)	1.38 (0.66-2.91)	<0.001
Primary endpoints						
Atrial fibrillation, n (%)	3892 (12.26)	1489 (18.77)	1075 (13.55)	863 (10.88)	465 (5.86)	<0.001
Heart failure, n (%)	3045 (9.60)	1346 (16.97)	790 (9.96)	627 (7.90)	282 (3.55)	<0.001
Cardiovascular mortality, n (%)	1911 (6.02)	770 (9.71)	532 (6.71)	395 (4.98)	214 (2.70)	<0.001
All-cause mortality, n (%)	5788 (18.24)	2048 (25.82)	1543 (19.45)	1311 (16.53)	886 (11.17)	<0.001

The chi-square test was used for the comparison of categorical variables; the t-test was employed to compare variates with normal distribution, and the rank-sum test was conducted for variates with skewed distribution. eGDR, estimated glucose disposal rate; BMI, body mass index; WC, waist circumference; SBP, systolic blood pressure; DBP, diastolic blood pressure; eGFR, estimated glomerular filtration rate; Ua, uric acid ; BUN, blood urea nitrogen; TC, total cholesterol; TG, HDL-C, high-density lipoprotein cholesterol; LDL-C,low-density lipoprotein cholesterol; GLU, glucose; Scr, serum creatinine; Cys-C,Cystatin C; ALB, Albumin; CRP, C-reactive protein.

### Analysis of the association of eGDR with AF, HF, and cardiovascular mortality

**Figure 2:**
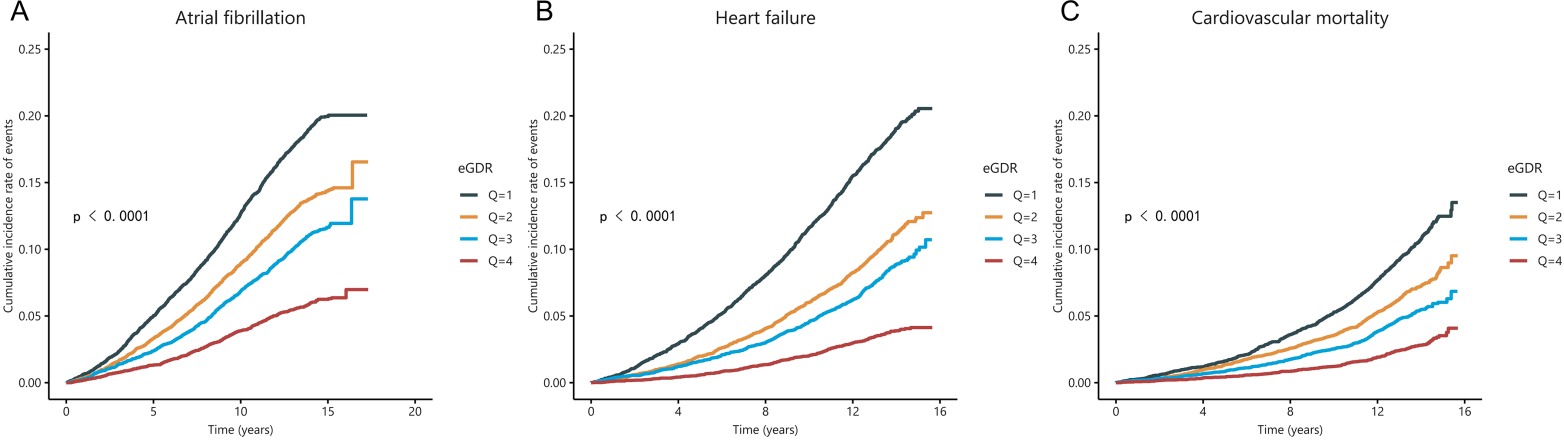
The Kaplan-Meier curve for cumulative incidence of AF **(A)**, HF **(B)**, and cardiovascular mortality **(C)** was based on eGDR quartiles for diabetic participants.

During a median follow-up of 12.8 years, 3,892 (12.26%) subjects developed atrial fibrillation (AF), 3,045 (9.60%) subjects developed heart failure (HF), and 1,911 (6.02%) subjects experienced cardiovascular mortality. The Kaplan-Meier survival curves and Log-rank test showed that the cumulative incidence of AF, HF and cardiovascular mortality was was significantly lower in the highest eGDR quartile compared to the lowest quartile (log-rank *P* < 0.001 for all) (**Figure 2**).

We conducted three Cox regression models to investigate the associations between eGDR with the risk of AF, HF and cardiovascular mortality. The results demonstrated that eGDR was significantly associated with both AF (HR=0.84, 95% CI: 0.83-0.85 in Model 1; HR = 0.90, 95% CI: 0.88-0.92 in Model 2; HR = 0.94, 95% CI: 0.91-0.96 in Model 3), HF (HR = 0.79, 95% CI: 0.78-0.80 in Model 1; HR = 0.82, 95% CI: 0.81-0.84 in Model 2; HR = 0.78, 95% CI: 0.74-0.82 in Model 3), and cardiovascular mortality (HR = 0.81, 95% CI: 0.80-0.83 in Model 1; HR = 0.84, 95% CI: 0.82-0.86 in Model 2; HR = 0.86, 95% CI: 0.83-0.88 in Model 3) (**Table 2**).

**Table 2:** HRs (95% CIs) for AF, HF, and cardiovascular mortality according to the eGDR quartiles.

Variables	Overall	Quartiles of eGDR				
		Q1 (<4.167)	Q2 (4.168-5.486)	Q3 (5.487-7.689)	Q4 (>7.690)	*P for trend*
AF						
Number of incidence	N=3892	N=1489	N=1075	N=863	N=465	
Model 1 HR (95%CI) P-value	0.84 (0.83, 0.85) <0.001	Reference	0.70 (0.65,0.76) <0.001	0.55 (0.51, 0.60) <0.001	0.29 (0.26, 0.32) <0.001	<0.001
Model 2 HR (95%CI) P-value	0.90 (0.88, 0.92) <0.001	Reference	0.83 (0.76, 0.90) <0.001	0.78 (0.70, 0.86) <0.001	0.53 (0.47, 0.60) <0.001	<0.001
Model 3 HR (95%CI) P-value	0.94 (0.91, 0.96) <0.001	Reference	0.86 (0.79, 0.94) 0.001	0.86 (0.78, 0.96) 0.007	0.70 (0.60, 0.82) <0.001	<0.001
HF						
Number of incidence	N=3045	N=1346	N=790	N=627	N=282	
Model 1 HR (95%CI) P-value	0.79 (0.788, 0.80) <0.001	Ref ( 1.0)	0.55 (0.50, 0.60) <0.001	0.43 (0.39, 0.47) <0.001	0.19 (0.16, 0.21) <0.001	<0.001
Model 2 HR (95%CI) P-value	0.82 (0.81, 0.83) <0.001	Ref ( 1.0)	0.60 (0.54, 0.66) <0.001	0.54 (0.48, 0.61) <0.001	0.30 (0.26, 0.35) 0.001	<0.001
Model 3 HR (95%CI) P-value	0.7 8 (0.74, 0.82) <0.001	Ref ( 1.0)	0.66 (0.59, 0.72) <0.001	0.65 (0.57, 0.73) <0.001	0.43 (0.35, 0.51) <0.001	<0.001
Cardiovascular mortality						
Number of incidence	N=1911	N=770	N=532	N=395	N=214	
Model 1 HR (95%CI) P-value	0.81 (0.80, 0.83) <0.001	Ref ( 1.0)	0.67 (0.60, 0.75) <0.001	0.49 (0.43, 0.55) <0.001	0.26 (0.22, 0.30) <0.001	<0.001
Model 2 HR (95%CI) P-value	0.84 (0.82, 0.86) <0.001	Ref ( 1.0)	0.69 (0.61, 0.78) <0.001	0.59 (0.51, 0.68) <0.001	0.39 (0.33, 0.47) 0.001	<0.001
Model 3 HR (95%CI) P-value	0.86 (0.83, 0.88) <0.001	Ref ( 1.0)	0.76 (0.67, 0.86) <0.001	0.69 (0.59, 0.80) <0.001	0.55 (0.42, 0.65) <0.001	<0.001

Model 1: Unadjusted.

Model 2: Adjusted for age, gender, race, education level, BMI, smoking status, and alcohol consumption status.

Model 3: Included all variables from Model 2, and further adjusted for SBP, DBP, TG, TC, eGFR, UA, aspirin, cholesterol-lowering medication, blood pressure medication, and insulin use.

Ref: reference; eGDR, estimated glucose disposal rate; AF, atrial fibrillation; HF, heart failure; BMI, body mass index; SBP, systolic blood pressure; DBP, diastolic blood pressure; TC, total cholesterol; TG, triglyceride; eGFR, estimated glomerular filtration rate; Ua, uric acid.

**Figure 3:**
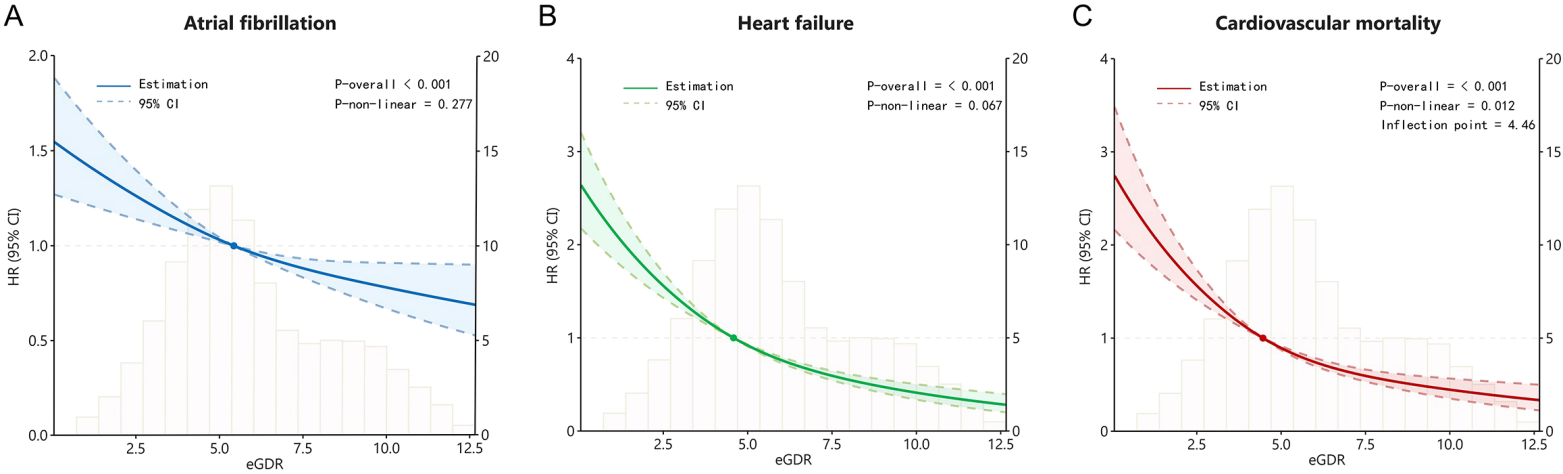
The restricted cubic spline curves for atrial fibrillation **(A)**, heart failure **(B)**, and cardiovascular mortality **(C)** based on eGDR for diabetic participants.

We performed restrictive cubic spline analysis based on the Cox proportional hazards regression model to investigate nonlinear correlations between eGDR and AF, HF and cardiovascular mortality. With eGDR as the x-axis and the hazard ratio as the y-axis, the smoothed curve fitting diagram after adjusting for confounding factors from Model 3 showed that eGDR presented a negative linear correlation with the AF (*P* for non-linear = 0.227) and HF (*P* for non-linearity = 0.067), and a non-linearity relationship with cardiovascular mortality (*P* for non-linearity = 0.012). Notably, despite the nonlinear pattern, an increasing level of eGDR was still associated with a decreasing trend in cardiovascular mortality (**Figure 3**).

### Joint association of eGDR and PRS with AF, HF and cardiovascular mortality

**Figure 4:**
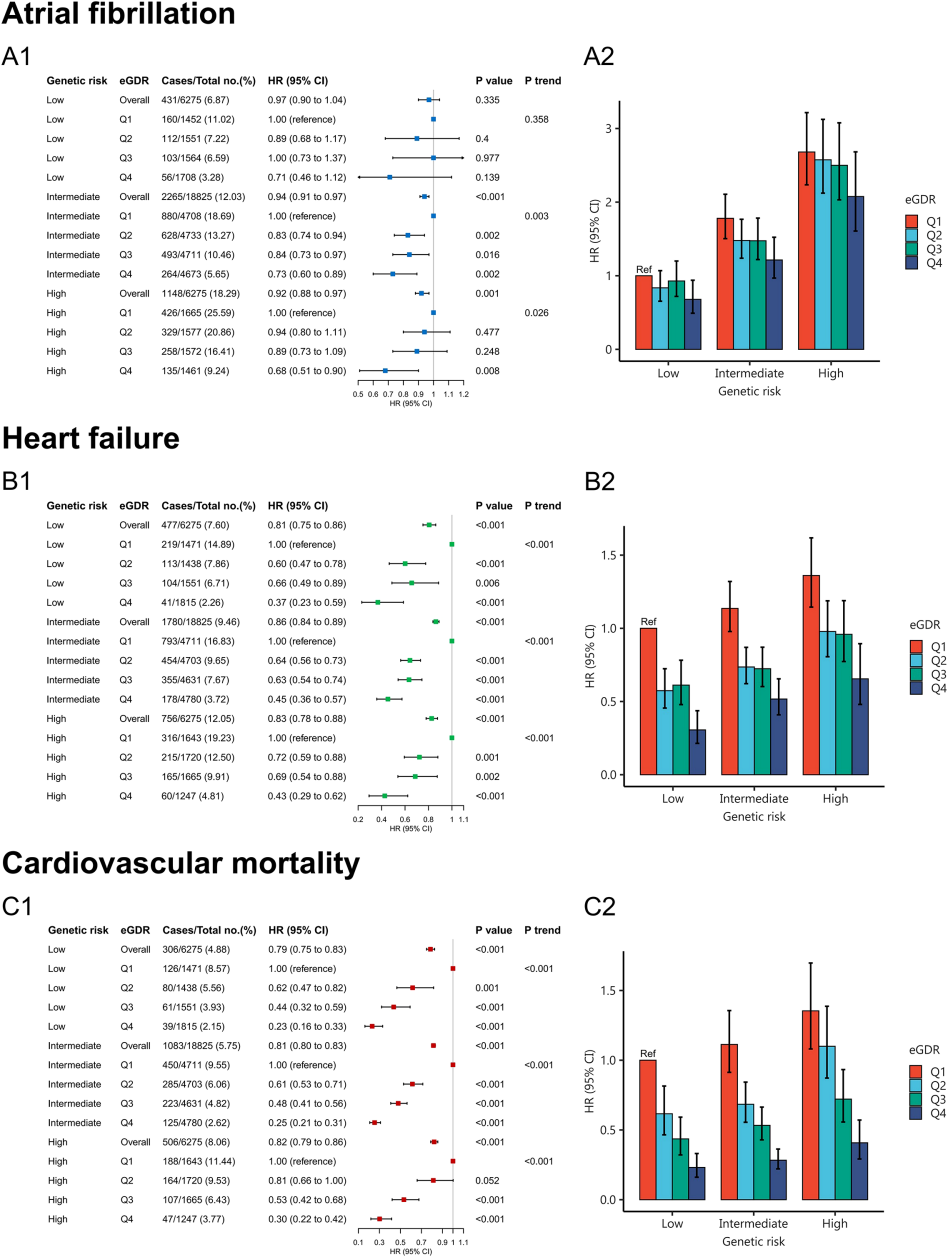
Joint association of eGDR and PRS with atrial fibrillation **(A1, A2)**, heart failure **(B1, B2)**, and cardiovascular mortality **(C1, C2)**.

Compared to low genetic risk, high genetic risk was associated with increased risk of AF (HR = 2.84, 95% CI:2.54-3.17), HF (HR = 1.55, 95% CI:1.38-1.74), and cardiovascular mortality (HR = 1.65, 95% CI:1.43-1.91), respectively (**Supplementary Table S1**). Behavioral and genetic factors jointly contributed to the risk of AF, HF and cardiovascular mortality. Therefore, we further investigated whether appropriate eGDR levels could mitigate the risks of AF, HF, and cardiovascular mortality in individuals with genetic susceptibility. **Figure 4A1** showed that no statistically significant difference between low genetic risk and eGDR for AF risk (*P* =0.335). However, high eGDR was associated with a 27% and 32% reduced risk of AF among intermediate (HR = 0.73, 95% CI: 0.60-0.89) and high genetic risk groups (HR = 0.68, 95% CI: 0.51-0.90), respectively. **Figure 4B1** showed that high eGDR was associated with a 63%, 55% and 53% reduced risk of HF among low (HR = 0.37, 95% CI: 0.23-0.59), intermediate (HR = 0.45, 95% CI: 0.36-0.57) and high genetic risk groups (HR = 0.47, 95% CI: 0.29-0.62), respectively. **Figure 4C1** showed that high eGDR was associated with a 77%, 75% and 70% reduced risk of cardiovascular mortality among low (HR = 0.23, 95% CI: 0.26-0.33), intermediate (HR = 0.25, 95% CI: 0.21-0.31) and high genetic risk groups (HR = 0.30, 95% CI: 0.22-0.42), respectively. Individuals with low eGDR and high genetic risk exhibited a increased risk of AF, HF, and cardiovascular mortality compared with those with high eGDR and low genetic risk (**Figures 4A2, B2, C2**; **Supplementary Table S2**).

### The variables that contribute most to model predictions

**Figure 5:**
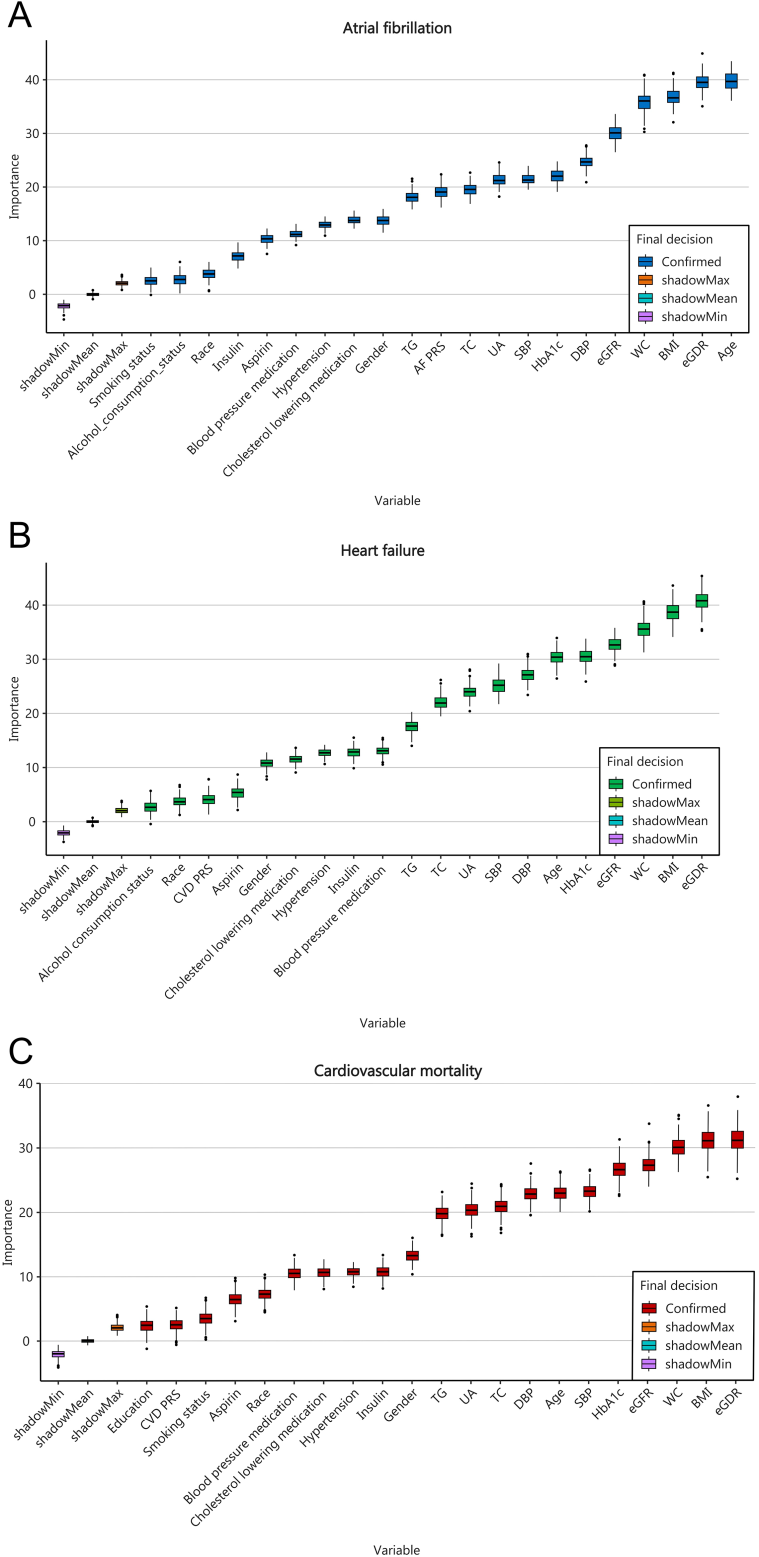
Feature selection based on the Boruta`s algorithm for AF **(A)**, HF **(B)**, and cardiovascular mortality **(C)**.

The feature screening results based on Boruta`s algorithm were showed in **Figure 5**; **Supplementary Figure S1**. After 500 iterations it was determined that the 20 variables most closely associated with AF were age, eGDR, BMI, WC, eGFR, DBP, SBP, HbAc1, UA, TC, AF-PRS, TG, gender, cholesterol-lowering medication, hypertension, blood pressure medication, asprin, insulin, race, alcohol consumption status, and smoking status. The 19 variables most closely associated with HF were eGDR, BMI, WC, eGFR, HbA1C, age, DSP, SBP, UA, TC, TG, blood pressure medication, insulin, hypertension, cholesterol-lowering medication, gender, asprin, CVD-PRS, and race. And the 20 variables most closely associated with cardiovascular mortality were eGDR, BMI, WC, eGFR, HbA1C, SBP, age, DSP, TC, UA, TG, gender, insulin, hypertension, cholesterol-lowering medication, blood pressure medication, race, asprin, smoking status, CVD-PRS, and education level. The analysis demonstrated that eGDR contributes most significantly to the the prediction of HF and cardiovascular mortality outcomes. While age contributes most significantly to the prediction of AF outcomes, eGDR ranks second in importance after age.

### Subgroup analysis

**Figure 6:**
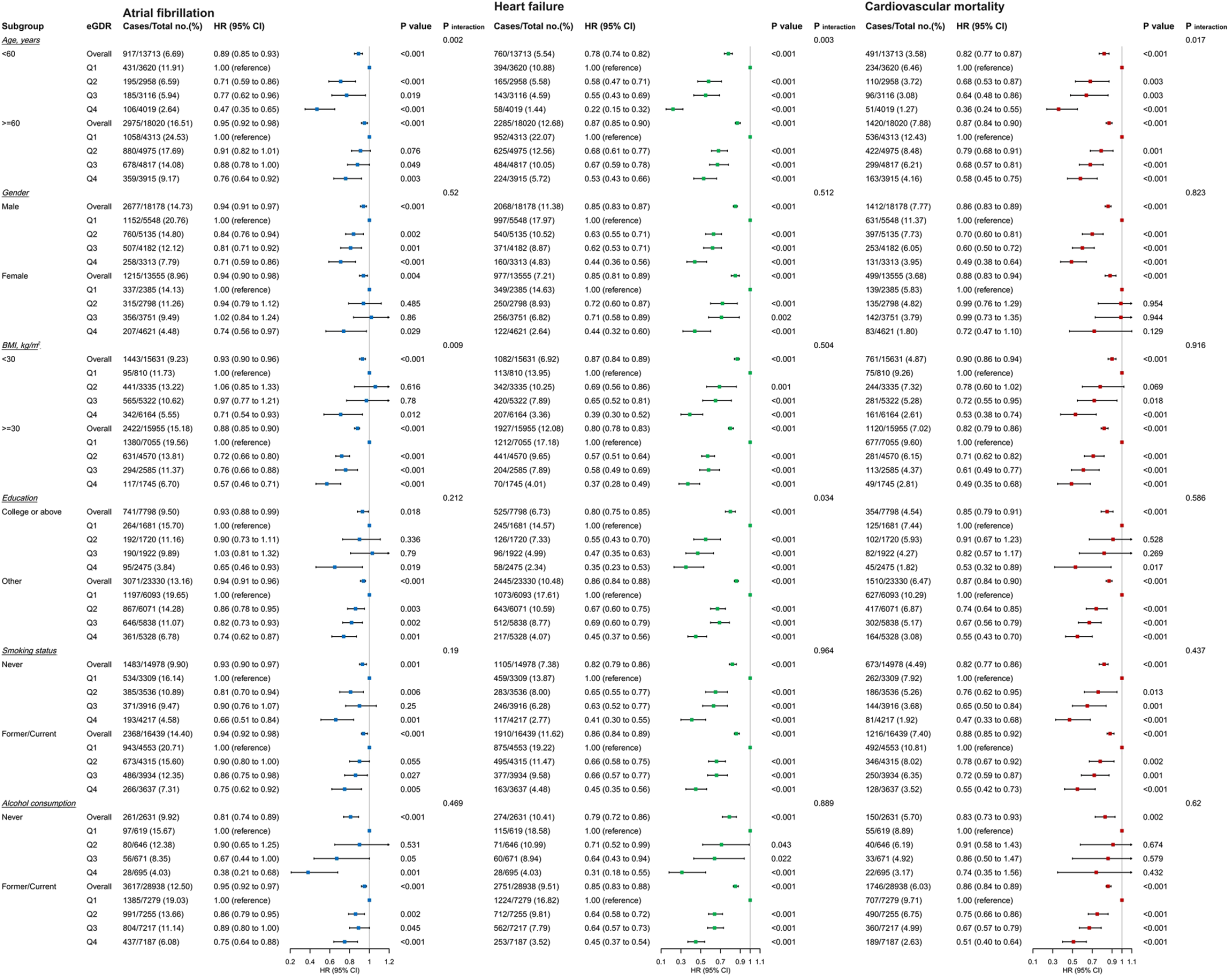
Subgroup and interaction analyses among the quartile Q1-Q4 and AF, HF, and cardiovascular mortality across various subgroups.

To further evaluate the effect of eGDR on outcome indicators, subgroup analyses was performed according to ages, gender, BMI, education level, smoking status, and alcohol consumption status. The subgroup analysis of AF (**Figure 6**) revealed that there was no significant interaction between most subgroups (gender, education level, smoking status, and alcohol consumption status) (*P* for interaction > 0.05). However, significant interactions were observed between eGDR and age as well as BMI subgroups. we found eGDR was strongly associated with AF incidence in the age subgroup (*P* for interaction = 0.002). Compared with the lowest eGDR, high eGDR was associated with a 53% reduced risk of AF incidence in subjects < 65 years (HR = 0.47, 95% CI: 0.35–0.65) and a 24% reduced risk in subjects ≥ 65 years (HR = 0.76, 95% CI: 0.64–0.92). In the BMI subgroup, an interaction was also observed between eGDR and AF incidence (*P* for interaction = 0.009). High eGDR was associated with a 29% reduced risk of AF incidence in subjects with BMI < 30 kg/m^2^ (HR = 0.71, 95% CI: 0.54-0.93) and a 43% reduced risk in subjects with BMI ≥ 30 kg/m^2^ (HR = 0.57, 95% CI: 0.46-0.71).

The subgroup analysis of heart failure (HF) (**Figure 6**) showed that there was no significant interaction between eGDR and most subgroups (gender, BMI, smoking status, and alcohol consumption status) (*P* for interaction > 0.05). However, a significant interaction was observed between eGDR and age (*P* for interaction < 0.001). High eGDR was associated with a 78% reduced risk of HF incidence in subjects < 65 years (HR = 0.22, 95% CI: 0.15-0.32) and a 47% reduced risk in subjects ≥ 65 years (HR = 0.53, 95% CI: 0.43-0.66).

The subgroup analysis of cardiovascular mortality (**Figure 6**) demonstrated that there was no significant interaction between eGDR and any subgroups (*P* for interaction > 0.05), except for the age subgroup (*P* for interaction = 0.017). High eGDR was associated with a 64% reduced risk of cardiovascular mortality in subjects < 65 years (HR = 0.36, 95% CI: 0.24-0.55) and a 42% reduced risk in subjects ≥ 65 years (HR = 0.58, 95% CI: 0.45-0.75).

### Mediation analysis

**Figure 7:**
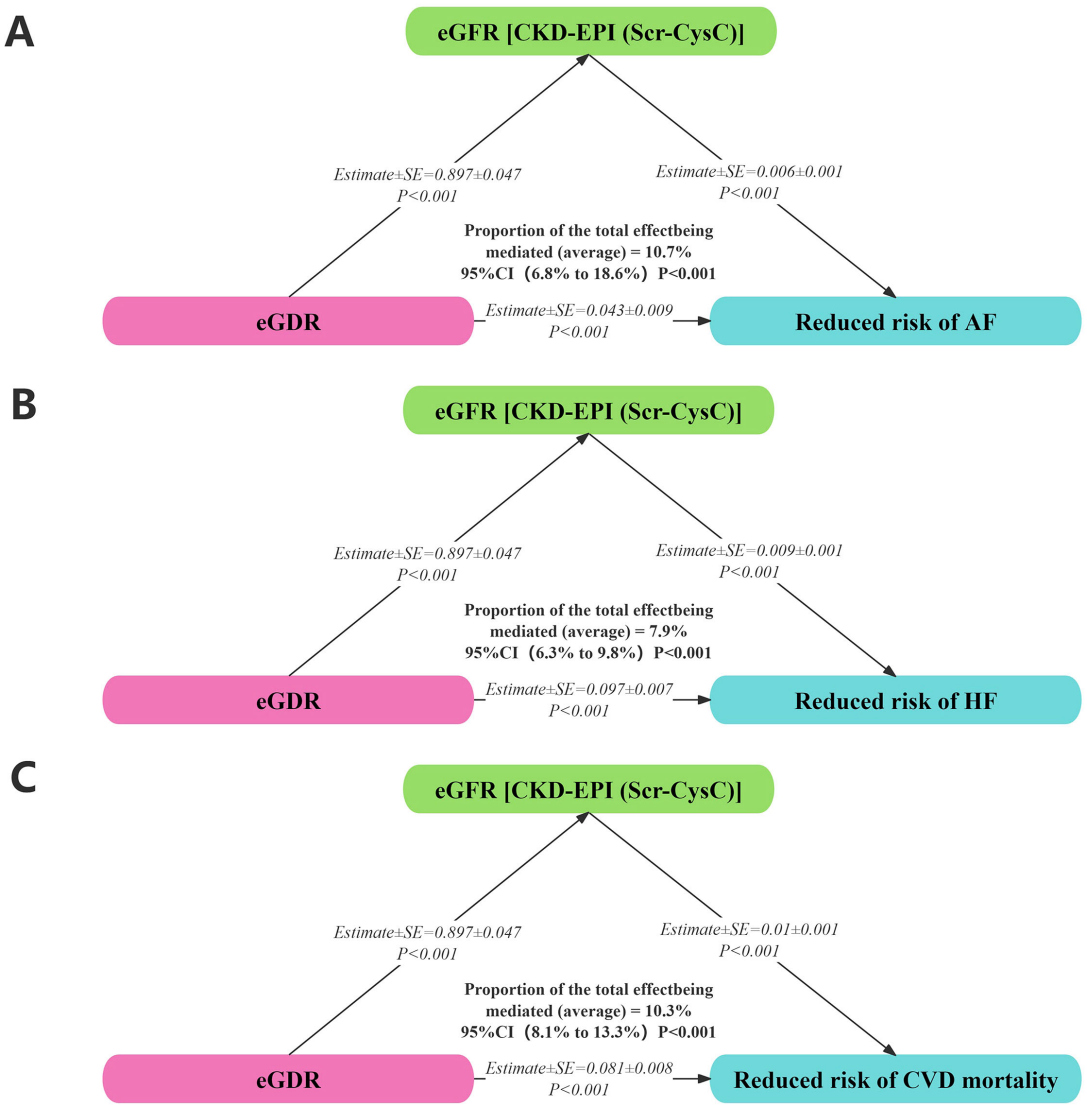
Mediation analysis on associations between eGDR with AF **(A)**, HF **(B)**, and cardiovascular mortality **(C)**.

Insulin resistance was associated with renal function, particularly in individuals with diabetes. Mediation analysis revealed that 10.7%, 7.9%, and 10.3% of the associations between eGDR and AF, HF, and cardiovascular mortality, respectively, among individuals with diabetes were mediated by eGFR. eGFR was positively correlated with a reduced risk of AF (Estimate ± SE = 0.006 ± 0.001, *P* < 0.001), HF (Estimate ± SE = 0.009 ± 0.001, *P* < 0.001), and cardiovascular mortality (Estimate ± SE = -0.010 ± 0.001, *P* < 0.001). Additionally, eGDR was positively correlated with eGFR (Estimate ± SE = 0.897 ± 0.047, *P* < 0.001) and a reduced risk of AF (Estimate ± SE = 0.043 ± 0.009, *P* < 0.001), HF (Estimate ± SE = 0.097 ± 0.007, *P* < 0.001), and cardiovascular mortality (Estimate ± SE = 0.081 ± 0.008, *P* < 0.001) (**Figure 7**).

## Discussion

This study clearly reveals a strong correlation between eGDR and atrial AF, HF, and cardiovascular mortality, providing important insights for a deeper understanding of the pathophysiology and clinical management of CVD. The following key findings were obtained: (1) Compared with the lowest eGDR group, the highest eGDR group exhibited a 30.1%, 57.2%, and 47.8% reduction in the risk of AF, HF, and cardiovascular mortality, respectively. eGDR demonstrated a linear relationship with AF and HF; as eGDR increased, the risks of AF and HF progressively decreased. Although eGDR was non-linearly associated with cardiovascular mortality, it exhibited a negative correlation. When eGDR was ≥ 4.46, it showed a protective effect on cardiovascular mortality in diabetic patients. (2) High eGDR was associated with a 27% and 32% reduced risk of AF in intermediate and high genetic risk groups, respectively, a 63%, 55%, and 57% reduced risk of HF in low, intermediate, and high genetic risk groups, respectively, and a 77%, 75%, and 70% reduced risk of cardiovascular mortality in low, intermediate, and high genetic risk groups, respectively. High eGDR could reduce the risk of AF, HF, and cardiovascular mortality in diabetic patients with high genetic susceptibility. (3) Boruta’s algorithm demonstrated that eGDR contributes most significantly to the prediction of AF, HF, and cardiovascular mortality outcomes. (4) Mediation analysis revealed that 10.7%, 7.9%, and 10.3% of the relationships between eGDR and AF, HF, and cardiovascular mortality, respectively, among individuals with diabetes were mediated by eGFR.

The impact of IR on the cardiovascular system is multidimensional. IR reduces cellular sensitivity to insulin, rendering glucose ineffective for cellular use, and prolongs hyperglycaemic states, which may cause a range of vascular lesions. This leads to lipid deposition on vascular walls and accelerates the progression of atherosclerosis. Some reports indicate that IR represents a chronic inflammatory state (21, 22). In the pathology of IR, adipose tissue secretes various inflammatory factors such as interleukin-6 (IL-6) and tumour necrosis factor-α (TNF-α) (23, 24). These inflammatory factors activate inflammatory cells such as monocytes and T-lymphocytes, which adhere to vascular endothelial cells and migrate to the subendothelium, where they phagocytose lipids to form foam cells, an early event in atherosclerotic plaque formation (25). Additionally, inflammatory factors inhibit the synthesis of nitric oxide, an important vasodilator, and a decrease in its synthesis leads to vascular endothelial dysfunction, promoting vasoconstriction and platelet aggregation, further increasing the risk of CVD (26).

A clear correlation between IR and hypertension has been demonstrated (27). On the one hand, insulin can directly act on renal tubules to increase sodium reabsorption; on the other hand, IR activates the sympathetic nervous system, increases catecholamine secretion, stimulates the renin-angiotensin-aldosterone system (RAAS), and contributes to increased aldosterone secretion (28, 29). These changes lead to sodium and water retention, resulting in high blood pressure. Chronic high blood pressure increases cardiac afterload, leading to myocardial hypertrophy, ventricular remodelling, and an increased risk of AF, HF, and cardiovascular mortality (30, 31). In addition, IR and insulin secretion are closely associated with the pancreas and gastrointestinal system (32, 33). Previous study had demonstrated a significant link between gastrointestinal disorders and CVD, particularly AF. Despite their apparent differences as distinct pathological phenomena, they in fact share common pathogenic mechanisms, thereby establishing interconnections (34). We found that, among diabetic patients with a BMI ≥ 30 kg/m^2^, an increase in eGDR was associated with a decreased risk of AF. Previous studies have demonstrated that obesity, IR, and excise are associated with the occurrence of AF, and however, the interrelationship among these three factors requires further investigation (35–37).

In past clinical practice, fasting plasma glucose (FPG), triglyceride-glucose index (TyG index), metabolic score for insulin resistance (METS-IR), and homeostasis model assessment of insulin resistance (HOMA-IR) were commonly used to evaluate IR (38–41). The TyG index was positively correlated with the incidence of CVD, all-cause mortality, and cardiovascular mortality in the general population (42). Previous studies have shown that METS-IR has important predictive value for coronary heart disease, hypertension, coronary artery calcification, diabetes, and non-alcoholic fatty liver disease, and higher levels of METS-IR indicate a higher degree of IR, placing individuals at higher risk of metabolic disorders (38, 43). HOMA-IR is calculated as (fasting plasma glucose × fasting plasma insulin)/22.5, serving as a quantifiable measure of IR (44). However, the complicated calculation formula and inconvenient detection method hindered its widespread use, and risk assessment for CVD in patients with diabetes was mainly based on traditional risk factors such as age, blood pressure, lipids, and blood glucose, which often do not comprehensively reflect the patient’s cardiovascular risk status. Several previous findings indicate that eGDR impacts the prognosis of CVD (45–47). Therefore, the potential application value of eGDR deserves in-depth exploration. Xing et al. (48) found that eGDR serves as a potential biomarker for CVD risk assessment as a comprehensive indicator of glucose metabolic status. A nationwide prospective cohort study in China indicated that sustained low eGDR was associated with an increased risk of new-onset CVD (HR = 2.51, 95% CI: 2.04-3.09) in middle-aged and elderly populations (49). Besides, recent studies suggested that low eGDR was associated with an increased risk of stroke (HR = 0.77, 95% CI: 0.69-0.87) and cardiovascular mortality (HR = 0.82, 95% CI: 0.70-0.95) in individuals with type 2 diabetes (50). Li et al. (51) found that eGDR could be a potential biomarker for predicting AF recurrence after ablation, and participants with an eGDR ≥ 8 mg/kg/min had a lower risk of AF recurrence than those with an eGDR < 4 mg/kg/min (HR = 0.28, 95% CI: 0.18-0.42).

Peng et al. proved that eGDR could be a promising tool for predicting cardiovascular comorbidities and mortality, and non-diabetic chronic kidney disease (CKD) patients with high eGDR levels had lower risks of CVD events (HR = 0.641, 95% CI: 0.559-0.734) (18). Therefore, we further investigated the association of eGDR with AF, HF, and cardiovascular mortality using mediation analysis and found that eGDR influences AF, HF, and cardiovascular mortality through eGFR (10.7%, 7.9%, and 10.3%, respectively) in patients with diabetes. However, how eGDR interacts with eGFR to influence the prognosis of cardiovascular disease remains unclear. The kidney plays a critical role in maintaining water-electrolyte balance and excreting metabolic wastes from the body. Sodium and water retention increases cardiac preload, places cardiomyocytes under long-term stress, and promotes myocardial remodelling (52, 53). Electrolyte disturbances, such as abnormalities in potassium and magnesium ion concentrations, affect the electrophysiological stability of cardiomyocytes and predispose individuals to atrial fibrillation (54). Activation of the RAAS is a key component of renal dysfunction affecting the cardiovascular system (55). After RAAS activation, angiotensin II production increases, causing vasoconstriction and elevated blood pressure, which further aggravates cardiac afterload and stimulates cardiomyocytes to become hypertrophic and fibrotic, accelerating the progression of CVD (56).

The latest research has shown that eGDR is inversely associated with the incidence of myocardial infarction (MI), HF, AF, and ischemic stroke in the general population, and it is believed that eGDR serves as a more valuable predictive indicator than TyG, TyG-WC, TyG-BMI, TyG-WHtR, TG/HDL-C, and METS-IR for CVD events in clinical practice (57). Zhang et al. found (58) that eGDR may have a linear and robust association with prevalent HF (*P* for non-linearity = 0.313) and a potential value in reflecting the prevalence of HF in the general population (AUC = 0.873, *P* = 0.008). Our conclusions are consistent with those of the previous study. However, we extended our analysis to examine the relationship between eGDR and AF, HF, and cardiovascular mortality using polygenic risk scores (PRS), Boruta’s algorithm, and mediation analysis, conducting a precise and systematic evaluation of the predictive value of eGDR for these diseases. We found that high eGDR could reduce the risk of AF, HF, and cardiovascular mortality in individuals with higher genetic risk among diabetic patients. However, these associations were not significant for AF in individuals with low genetic risk. This may result from a synergistic interaction between genes and the metabolic environment, and the causal relationship between eGDR and AF requires further in-depth studies. Integration of eGDR and PRS may optimize cardiovascular risk stratification. In individuals with high PRS, early monitoring of eGDR and intervention of IR may hold significant value in the prevention of CVD. The PRS data originated from the UK Biobank (UKB), and 31,375 cases were included. Therefore, we believe that the application of eGDR in predicting the risk of AF, HF, and cardiovascular mortality in patients with diabetes has high credibility.

## Strengths and limitations

There were some limitations to this study. First, although the sample size reflects the research question to some extent, it was still relatively limited and may not cover all possible clinical situations and population characteristics, potentially introducing bias into the study results. Larger multi-centre studies are needed in the future to further validate and refine our findings, improving the reliability and universality of the findings. Second, this study was a observational prospective cohort study, and although we attempted to control for confounding factors, there may still be unmeasured or incompletely corrected factors that could affect the relationship between eGDR and CVD outcomes. Additionally, the calculation of eGDR in this study was based on specific formulas and laboratory indices, and different testing methods may have certain effects on eGDR values. Finally, the participants were predominantly from European populations, limiting the consistency and comparability with other populations. Nevertheless, it retains a certain degree of reference value.

## Conclusions

In conclusion, despite certain limitations in this study, the identification of the negative correlation between eGDR and the risks of AF, HF, and cardiovascular mortality among diabetic participants in the UKB holds great significance. eGDR has shown remarkable potential in predicting these critical cardiovascular outcomes in diabetic patients. In the future, it will be necessary to further investigate its underlying molecular mechanisms and conduct large-scale, multi-centre, prospective clinical studies to comprehensively explore the value of eGDR in the diagnosis, treatment, and prognosis of CVD. This will provide stronger evidence for the precise prevention and management of CVD in patients with diabetes.

## Data Availability

The datasets presented in this study can be found in online repositories. The names of the repository/repositories and accession number(s) can be found below: https://www.ukbiobank.ac.uk/, 106027.
