# Supplementary material for: Association of estimated glucose disposal rate with atrial fibrillation, heart failure and cardiovascular mortality in patients with diabetes: a prospective cohort study from the UK Biobank
Source: Front Endocrinol (Lausanne). 2025 Jul 18;16:1579836. doi: 10.3389/fendo.2025.1579836 (PMC12313486; doi:10.3389/fendo.2025.1579836)
Supplement: Supplementary file 1 [file DataSheet1.docx]

| **Table S1 The associations between PRS and risk of AF, HF, and cardiovascular mortality** | | | | | | | | | | |
| --- | --- | --- | --- | --- | --- | --- | --- | --- | --- | --- |
| Variables PRS Atrial fibrillation Heart failure Cardiovascular mortality  *HR(95%CI)* *P-value P for trend* *HR(95%CI)* *P-value P for trend HR(95%CI)*  *P-value P for trend* | | | | | | | | | | |
| Model1 | Overall | 1.10 (1.06,1.15) | <0.001 |  | 1.22 (1.18,1.27) | <0.001 |  | 1.24 (1.18,1.30) | <0.001 |  |
|  | low | Ref |  | <0.001 | Ref |  | <0.001 | Ref |  | <0.001 |
|  | intermediate | 1.80 (1.63,2.00) | <0.001 |  | 1.26 (1.14,1.40) | <0.001 |  | 1.19 (1.05,1.35) | 0.008 |  |
|  | high | 2.85 (2.55,3.18) | <0.001 |  | 1.65(1.47,1.85) | <0.001 |  | 1.69 (1.47,1.95) | <0.001 |  |
| Model2 | Overall | 1.49 (1.44,1.55) | <0.001 |  | 1.24 (1.20,1.29) | <0.001 |  | 1.26 (1.21,1.32) | <0.001 |  |
|  | low | Ref |  | <0.001 | Ref |  | <0.001 | Ref |  | <0.001 |
|  | intermediate | 1.76 (1.59,1.95) | <0.001 |  | 1.30 (1.20,1.41) | <0.001 |  | 1.22 (1.07,1.38) | 0.002 |  |
|  | high | 2.90 (2.59,3.24) | <0.001 |  | 1.88 (1.68,2.11) | <0.001 |  | 1.79 (1.55,2.06) | <0.001 |  |
| Model3 | Overall | 1.08 (1.02,1.14) | 0.011 |  | 1.20(1.15,1.24) | <0.001 |  | 1.23 (1.17,1.28) | <0.001 |  |
|  | low | Ref |  | <0.001 | Ref |  | <0.001 | Ref |  | <0.001 |
|  | intermediate | 1.73 (1.56,1.92) | <0.001 |  | 1.23 (1.11,1.36) | <0.001 |  | 1.18 (1.04,1.34) | 0.010 |  |
|  | high | 2.84 (2.54,3.17) | <0.001 |  | 1.55 (1.38,1.74) | <0.001 |  | 1.65 (1.43,1.91) | <0.001 |  |

Model 1: Unadjusted

Model 2: Adjusted for age, gender, race, education level, BMI, smoking status, and alcohol consumption status

Model 3: included all variables from Model 2, and further adjusted for SBP, DBP, TG, TC, eGFR, UA, aspirin, cholesterol-lowering medication, blood pressure medication, and insulin use

Abbreviations: Ref: reference; PRS: polygenic risk score; eGDR: estimated glucose disposal rate; BMI: body mass index; SBP: systolic blood pressure; DBP: diastolic blood pressure; TC: total cholesterol; TG: triglyceride; eGFR: estimated glomerular filtration rate; Ua: uric acid

| **Table S2 Joint association of eGDR and PRS with AF, HF and cardiovascular mortality** | | | | | | | |
| --- | --- | --- | --- | --- | --- | --- | --- |
| Genetic risk eGDR Atrial fibrillation Heart failure Cardiovascular mortality  *HR(95%CI)* *P-value*  *HR(95%CI)* *P-value HR(95%CI)*  *P-value* | | | | | | | |
| Low | Q1 | Ref |  |  |  |  |  |
| Low | Q2 | 0.86 (0.65,1.85) | 0.150 | 0.57 (0.46,0.72) | <0.001 | 0.62 (0.47,0.82) | <0.001 |
| Low | Q3 | 0.93 (0.72,1.20) | 0.570 | 0.61 (0.48,0.78) | <0.001 | 0.44 (0.32,0.59) | <0.001 |
| Low | Q4 | 0.68 (0.49,0.94) | 0.020 | 0.31 (0.21,0.44) | <0.001 | 0.23 (0.16,0.33) | <0.001 |
| intermediate | Q1 | 1.78 (1.50,2.11) | <0.001 | 1.14 (0.98,1.32) | 0.100 | 1.11 (0.91,1.36) | 0.290 |
| intermediate | Q2 | 1.48 (1.24.1.77) | <0.001 | 0.74(0.62,0.87) | <0.001 | 0.68 (0.56,0.84) | <0.001 |
| intermediate | Q3 | 1.48 (1.22,1.78) | <0.001 | 0.72 (0.60,0.87) | <0.001 | 0.53 (0.43,0.66) | <0.001 |
| intermediate | Q4 | 1.21 (0.97,1.52) | 0.090 | 0.52 (0.41,0.66) | <0.001 | 0.28 (0.22,0.36) | <0.001 |
| high | Q1 | 2.68 (2.24,3.32) | <0.001 | 1.36 (1.15,1.62) | <0.001 | 1.35 (1.08,1.70) | 0.010 |
| high | Q2 | 2.58 (2.12,3.12) | <0.001 | 0.98 (0.81,1.19) | 0.830 | 1.10 (0.87,1.39) | 0.420 |
| high | Q3 | 2.50 (2.03,3.08) | <0.001 | 0.96(0.77,1.19) | 0.700 | 0.72 (0.56,0.93) | 0.010 |
| high | Q4 | 2.08 (1.61,2.68) | <0.001 | 0.66 (0.48,0.90) | 0.010 | 0.41 (0.29,0.57) | <0.001 |

Abbreviations: Ref: reference; eGDR: estimated glucose disposal rate; PRS: polygenic risk score; AF: atrial fibrillation; HF: heart failure

| 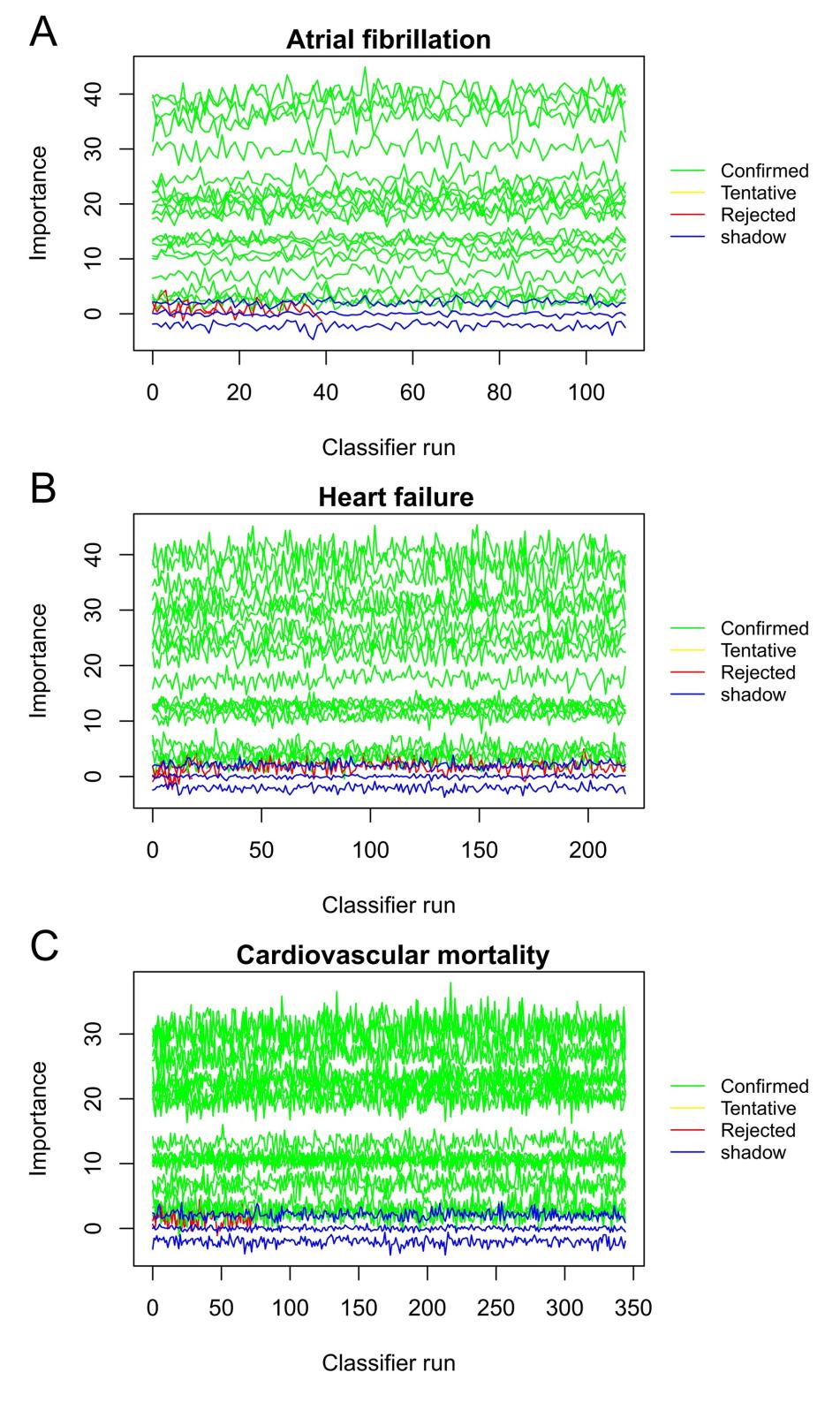 |
| --- |
| Fig. S1 The value evolution of Z-score in the screening process for atrial fibrillation (A), heart failure (B), and cardiovascular mortality (C). In A, B, and C, the horizontal axis represents the number of iterations, and the vertical axis represents the change in Z-values during the screening process. |
